# Early deficits in insulin secretion, beta cell mass and islet blood perfusion precede onset of autoimmune type 1 diabetes in BioBreeding rats

**DOI:** 10.1007/s00125-017-4512-z

**Published:** 2017-12-06

**Authors:** Anya Medina, Saba Parween, Sara Ullsten, Neelanjan Vishnu, Yuk Ting Siu, My Quach, Hedvig Bennet, Alexander Balhuizen, Lina Åkesson, Nils Wierup, Per Ola Carlsson, Ulf Ahlgren, Åke Lernmark, Malin Fex

**Affiliations:** 1 Lund University Diabetes Centre, Clinical Research Centre, Skåne University Hospital (SUS), Jan Waldentrömsgata 35, SE-20502 Malmö, Sweden; 2 Umeå Centre for Molecular Medicine, Umeå University, Umeå, Sweden; 3 Medical Cell Biology, Uppsala Biomedical Centre, Uppsala, Sweden

**Keywords:** Beta cell dysfunction, Beta cell mass, Insulin secretion, Islet blood flow, Type 1 diabetes

## Abstract

**Aims/hypothesis:**

Genetic studies show coupling of genes affecting beta cell function to type 1 diabetes, but hitherto no studies on whether beta cell dysfunction could precede insulitis and clinical onset of type 1 diabetes are available.

**Methods:**

We used 40-day-old BioBreeding (BB) DR*Lyp/Lyp* rats (a model of spontaneous autoimmune type 1 diabetes) and diabetes-resistant DR*Lyp/+* and DR*+/+* littermates (controls) to investigate beta cell function in vivo, and insulin and glucagon secretion in vitro*.* Beta cell mass was assessed by optical projection tomography (OPT) and morphometry. Additionally, measurements of intra-islet blood flow were performed using microsphere injections. We also assessed immune cell infiltration, cytokine expression in islets (by immunohistochemistry and qPCR), as well as islet *Glut2* expression and ATP/ADP ratio to determine effects on glucose uptake and metabolism in beta cells.

**Results:**

DR*Lyp/Lyp* rats were normoglycaemic and without traces of immune cell infiltrates. However, IVGTTs revealed a significant decrease in the acute insulin response to glucose compared with control rats (1685.3 ± 121.3 vs 633.3 ± 148.7; *p* < 0.0001). In agreement, insulin secretion was severely perturbed in isolated islets, and both first- and second-phase insulin release were lowered compared with control rats, while glucagon secretion was similar in both groups. Interestingly, after 5–7 days of culture of islets from DR*Lyp/Lyp* rats in normal media, glucose-stimulated insulin secretion (GSIS) was improved; although, a significant decrease in GSIS was still evident compared with islets from control rats at this time (7393.9 ± 1593.7 vs 4416.8 ± 1230.5 pg islet^−1^ h^−1^; *p* < 0.0001). Compared with controls, OPT of whole pancreas from DR*Lyp/Lyp* rats revealed significant reductions in medium (4.1 × 10^9^ ± 9.5 × 10^7^ vs 3.8 × 10^9^ ± 5.8 × 10^7^ μm^3^; *p* = 0.044) and small sized islets (1.6 × 10^9^ ± 5.1 × 10^7^ vs 1.4 × 10^9^ ± 4.5 × 10^7^ μm^3^; *p* = 0.035). Finally, we found lower intra-islet blood perfusion in vivo (113.1 ± 16.8 vs 76.9 ± 11.8 μl min^−1^ [g pancreas]^−1^; *p* = 0.023) and alterations in the beta cell ATP/ADP ratio in DR*Lyp/Lyp* rats vs control rats.

**Conclusions/interpretation:**

The present study identifies a deterioration of beta cell function and mass, and intra-islet blood flow that precedes insulitis and diabetes development in animals prone to autoimmune type 1 diabetes. These underlying changes in islet function may be previously unrecognised factors of importance in type 1 diabetes development.

**Electronic supplementary material:**

The online version of this article (10.1007/s00125-017-4512-z) contains peer-reviewed but unedited supplementary material, which is available to authorised users.

## Introduction

Type 1 diabetes is associated with the immune-mediated destruction of islet beta cells. Studies in human monozygotic twins, sharing identical genomes, demonstrate pairwise type 1 diabetes of 13–52%, suggesting that environmental and genetic causes may contribute similarly to the disease [1].

Research pertaining to the genetic contribution of type 1 diabetes have for the past decades focused on genetic loci implicated in regulation and selection of autoreactive T lymphocytes [2], although single nucleotide polymorphisms within the human insulin (*INS*) gene (mainly present in beta cells) remain one of the most important risk factors for the development of type 1 diabetes [3]. Recent studies have revealed that several candidate genes found in genome-wide association studies of type 1 diabetes susceptibility loci are expressed in beta cells and could thus influence beta cell function [4].

The BioBreeding (BB; LEW.1WR1) rat acts as a model of type 1 diabetes, whereby type 1 diabetes is suggested to originate from selective autoimmune destruction of beta cells [5]. As in humans, the major histocompatibility complex holds genetic factors that predict disease in this model [6, 7]. This explains some, but not all, of the inherited predisposition to type 1 diabetes. In the inbred BB rat strain BBDR*Lyp/Lyp* (herein referred to as DR*Lyp/Lyp*), onset of type 1 diabetes is linked to lymphopaenia, which is caused by a frameshift mutation in the *Gimap5* gene, while their littermates DR*Lyp/+* and DR*+/+* are resistant to diabetes [8, 9]. Loss of T cells because of lymphopaenia affects both CD4^+^ and CD8^+^ T cells, especially ART2.1^+^ T cells [5]. In fact, depletion of the ART2.1^+^ T cells in diabetes-resistant BB rats induces type 1 diabetes, suggesting that loss of regulatory T cells is associated with insulitis and type 1 diabetes [10].

Early changes in beta cell function and blood glucose have not been elucidated in DR*Lyp/Lyp* rats, although local changes in beta cells in inbred DR*Lyp/Lyp* are reflected by production of eotaxin (an eosinophil and mast cell recruiting factor) in islets at about 40 days of age, before insulitis, hyperglycaemia and type 1 diabetes [11, 12]. However, positive staining of infiltrating monocytes remains to be shown at this age [11]. Additionally, islets from 40-day-old DR*Lyp/Lyp* animals express lower levels of genes involved in the metabolism of reactive oxygen species (ROS) [13] and are more sensitive to changes in redox balance [14]. Over time, such an inherent sensitivity could contribute to accumulation of the ROS that diminish beta cell function, rendering cells more sensitive to immune cell attack.

Islet function is also dependent on functional islet vasculature and blood flow. In fact, inflammatory changes in vascular endothelial cells, characterised by increased expression of surface receptors, facilitate immune cell extravasation into the inflamed tissue [15]. Additionally, islet vasculature plays a critical role in maintaining oxygen and nutrient supply to the islets [16] and poor intra-islet blood flow is associated with changes in acute insulin response to glucose in vivo [17]. Interestingly, venular defects were observed in islets from BB (DP-BB/Wor) rats [18]. This, in combination with an underlying beta cell defect, could impair beta cell function and promote insulitis and beta cell destruction.

Currently, evidence of changes in beta cell function prior to onset of type 1 diabetes is limited. Therefore, we set out to explore whether insufficient beta cell function, or changes in beta cell mass and intra-islet blood flow, precede type 1 diabetes using the DR*Lyp/Lyp* rat as a disease model.

## Methods

### Animals

The BB rat was originally derived from a Canadian colony of outbred Wistar rats (originating from the Ottawa Health Research Institute, University of Ottawa, Ottawa, ON, Canada) that spontaneously develop hyperglycaemia and ketoacidosis, characteristics of clinical onset of type 1 diabetes. Heterozygous BB DR*Lyp/+* rats were used to obtain congenic DR*Lyp/Lyp* rats as previously described [9, 19]. Briefly, the *Lyp* region from diabetes-prone BB rats was introgressed onto the diabetes-resistant BB rat and kept in sibling breeding for more than 50 generations by heterozygous breeders to yield 25% DR*Lyp/Lyp*, 25% DR+/+ and 50% DR*Lyp*/+ rats. All DR*Lyp/Lyp* rats developed diabetes after transferring the entire colony from University of Washington, Seattle to Lund University (including the Clinical Research Centre in Malmö, Sweden), in 2008. Animals were bred/kept in a pathogen-free environment at the Clinical Research Centre in Malmö, Sweden. They were housed at 21–23°C (12 h light/dark cycle) and fed ad libidum. All experiments were approved by the Animal Ethical Committee in Uppsala and Lund. All animals used in experiments were 40 days old unless otherwise stated.

### Genotyping

Tail snips were obtained from rat pups between 25–30 days of age. DNA was isolated and genotyped based on microsatellite analysis, as previously described [9, 20].

### Blood glucose and plasma insulin levels

Blood glucose was tested daily at 08:00 hours in DR*Lyp/Lyp* (*n =* 225, 129 male [M]/96 female [F]) and control rats (DR*Lyp/+* and DR*+/+*; *n =* 100, 50M/50F) from day 37 (ELTE XL glucometer; Bayer Diabetes Care, Tarrytown, NY, USA). Animals were considered to have developed diabetes when blood glucose levels were >11.1 mmol/l for two consecutive days. Serum insulin was measured in a baseline group at 37–41 days of age (DR*Lyp/Lyp*: *n =* 7, 4M/3F; control rats: *n =* 10, 5M/5F), at 50 days (DR*Lyp/Lyp*: *n =* 6, 3M/3F; control rats: *n =* 10, 5M/5F), at 60 days (DR*Lyp/Lyp*: *n =* 6, 3M/3F; control rats: *n =* 11, 6M/5F) and at type 1 diabetes onset (DR*Lyp/Lyp*: *n =* 7, 4M/3F; control rats: *n =* 9, 5M/4F) in 10 μl of serum (rat insulin ELISA, Mercodia, Uppsala, Sweden). Blood was obtained from venipuncture of the tail vein in the fed state.

### IVGTT

Glucose (1 g/kg) (Sigma Aldrich, Stockholm, Sweden) was injected into the tail vein of DR*Lyp/Lyp* (*n =* 10, 6M/4F) and control (*n =* 10, 6M/4F) rats after 6 h of fasting. Blood samples were collected from the sublingual vein at 0, 1, 5, 10, 20, 50 and 75 min. Plasma glucose and insulin levels were measured (Infinity Glucose Oxidase Liquid Stable Reagent, Thermo Scientific, Waltham, MA, USA and Rat Insulin ELISA, Mercodia, respectively).

### Perifusion of isolated islets

Islets from DR*Lyp/Lyp* (*n =* 14, 9M/5F) and control rats (*n =* 8, 4M/4F) were isolated using collagenase digestion and incubated in RPMI-1640 medium containing 11.1 mmol/l glucose (Sigma Aldrich) + 10% FBS overnight at 37°C. Seventy islets per chamber were used in perifusion experiments (Suprafusion 1000 System; Brandel, Glasgow, UK). Islets were perifused with secretion assay buffer (SAB) containing: 114 mmol/l NaCl, 4.7 mmol/l KCl, 1.2 mmol/l KH_2_PO_4_, 1.16 mmol/l MgSO_4_, 25.5 mmol/l NaHCO_3_, 20 mmol/l HEPES, 2.5 mmol/l CaCl_2_ and 0.2% BSA (fatty acid free) (pH 7.2), supplemented with 2.8 mmol/l glucose for 2 h prior to sampling. Consecutive samples were taken at 2.8 mmol/l glucose to determine basal insulin release before challenging islets with a high glucose concentration (16.7 mmol/l). Experiments were concluded by estimating maximal insulin response by the addition of SAB containing 35 mmol/l KCl. The flow rate was 0.1 ml/min and temperature was kept at 37°C. Each fraction of perifusate was collected at 4 min intervals and stored at −20°C until analysed (Rat Insulin ELISA, Mercodia).

### Batch incubation of isolated islets of Langerhans

Isolated islets from DR*Lyp/Lyp* and control rats were cultured overnight (RPMI-1640 medium, 11.1 mmol/l glucose, 10% FBS [Sigma Aldrich]; DR*Lyp/Lyp*: *n =* 6, 3M/3F; controls: *n =* 6, 3M/3F), or for 5–7 days (RPMI medium, 5.6 mmol/l glucose, 10% FBS + penicillin [100 units/ml]–streptomycin [100 μg/ml]; DR*Lyp/Lyp*: *n =* 6, 3M/3F; controls: *n =* 7, 3M/4F) at 37°C, 5% CO_2_. Groups of three islets were placed in a well of a 96-well plate with SAB containing either 2.8 mmol/l or 16.7 mmol/l glucose at 37°C, 5% CO_2_. Experiments were performed with 6–8 replicates for each condition. Insulin and glucagon levels were determined after 1 h (Rat Insulin ELISA and Glucagon ELISA, respectively; Mercodia).

### Insulin content

Total insulin was extracted from 50 islets per animal (DR*Lyp/Lyp*: *n =* 6, 3M/3F; controls: *n =* 6, 3M/3F) using acid ethanol (0.18 mmol/l HCl in 95% ethanol). Extracted insulin was diluted and total insulin was measured (Rat Insulin ELISA; Mercodia).

### qPCR of islets of Langerhans

Isolated islets from DR*Lyp/Lyp* (*n =* 6, 3M/3F) and control (*n =* 7, 3M/4F) rats were frozen (−80°C) after isolation or after 5–7 days in culture (37°C, 5% CO_2_ in RPMI medium, 5.6 mmol/l glucose, 10% FBS + penicillin [100 units/ml]–streptomycin [100 μg/ml]). Total RNA was extracted (RNAeasy RNA purification kit; Qiagen, Hilden, Germany) and equal quantities of RNA were reverse transcribed (RevertAid First-Strand cDNA synthesis kit; Fermentas, Vilnius, Lithuania). mRNA levels were quantified (Maxima Probe/ROX qPCR Master Mix; Fermentas, Thermo Scientific, Helsingborg, Sweden) using an ABI PRISM 7900 (Applied Biosystems ViiA Real Time PCR System; Life Technologies, Foster City, CA, USA), using probes for *Il1b* (ID no. Rn00580432), *Tnf-α* (also known as *Tnf*) (ID no. Rn01525859), *Ifng* (ID no. Rn00594078) and *Glut2* (also known as *Slc2a2*) (ID no. Rn00563565) (Applied Biosystems). Samples were run in triplicate and the transcript quantity was normalised to the geometric mean of mRNA levels of the reference genes (Applied Biosystems) *Ppia* (ID no. Rn00690933), *Polr2a* (ID no. Rn01752026) and *Hprt* (also known as *Hprt1*) (ID no. Rn01527840), using the formula 2^(minCt – sampleCt)^.

### Blood flow measurements and islet morphometry

DR*Lyp/Lyp* (*n =* 11, 4M/7F) and control (*n =* 15, 6M/9F) rats were anaesthetised (i.p. injection of thiobutabarbital sodium; 120 mg/kg; Inactin; Sigma Aldrich) and placed on a heating pad to maintain body temperature. The trachea was detached and a polyethylene catheter was inserted to secure free airways. Catheters were inserted into the right ascending aorta and the left femoral artery. A pressure transducer was connected to the ascending aorta catheter. A blood sample was taken for blood glucose measurement (Freestyle Lite; Abbott, Calameda, CA, USA). When blood pressure had stabilised (10–15 min), animals were injected with 1.5 × 10^5^ microspheres (diameter: 10 μm) (E-Z Trac Ultraspheres; Stason Labs, Irwin, CA, USA) into the ascending aorta and blood was collected as described [21]. Animals were then euthanised and the pancreas and adrenal glands were dissected, weighed, cut in pieces and placed between object glasses. Object glasses containing pancreatic tissue were freeze–thawed to visualise islets [21]. The percentage of islet volume was determined by a point-counting [22], and the number of microspheres in the exocrine and endocrine pancreas, adrenal glands and reference sample was counted in a bright and dark field illumination microscope.

### Optical projection tomography imaging and quantification of islet beta cell distribution

Following euthanisation using CO_2_, pancreases from DR*Lyp/Lyp* (*n =* 6, 4M/2F) and control (*n =* 4, 2M/2F) rats were excised and processed for optical projection tomography (OPT) imaging [23]. Antibodies used for whole mount immunohistochemistry were: guinea pig anti-insulin (1:500; A0564; DAKO Denmark, Glostrup, Denmark) and IRDye 680 goat anti-guinea pig (1:250; 926-68077; LI-COR Biosciences, Lincoln, NE, USA). Pancreatic lobes were scanned individually using a near-infrared OPT setup equipped with a 665/45 excitation and a 725/50 emission filter (Chroma). Beta cell volumes were reconstructed based on the signal from insulin-specific antibodies and pseudo-coloured to highlight the distribution of small <1 × 10^6^ μm^3^ (white), medium 1 × 10^6^ μm^3^ to 5 × 10^6^ μm^3^ (yellow) and large >5 × 10^6^ μm^3^ (red) islets [23, 24].

### Live single cell ATP/ADP ratio measurements

Single cell ATP/ADP ratio measurements in islets from DR*Lyp/Lyp* (*n =* 91 islets) and control rats (*n =* 70 islets) were performed using the ATP biosensor, Perceval (Addgene, Cambridge, MA, USA). Islets were transduced [25, 26], plated and incubated on poly-d-lysine coated 8-well chambered cover glasses (Thermo Scientific, Waltham, MA, USA) for 2 h with RPMI medium + penicillin (100 units/ml)–streptomycin (100 μg/ml) containing the Perceval adenovirus. Fresh medium was added and cells were incubated overnight. The following day, cells were pre-incubated at 37°C in 400 μl buffer P (135 mmol/l NaCl, 3.6 mmol/l KCl, 1.5 mmol/l CaCl_2_, 0.5 mmol/l MgSO_4_, 0.5 mmol/l Na_2_HPO_4_, 10 mmol/l HEPES, 5 mmol/l NaHCO_3_, pH 7.4) containing 2.8 mmol/l glucose for 1.5 h. After this, cells were first imaged in the presence of low (2.8 mmol/l) glucose and then in the presence of high glucose (16.7 mmol/l) to investigate the basal and stimulated ATP/ADP ratio. Thereafter, ATP synthesis was inhibited by the addition of the ATP synthase inhibitor oligomycin (0.02 mg/ml) and an ionophore that uncouples ATP synthesis, carbonyl cyanide-*p*-trifluoromethoxyphenylhydrazone (FCCP; 0.05 mmol/l). Cells were imaged using 490 nm excitation and 520 nm emission filter settings on a Zeiss LSM510 inverted confocal fluorescence microscope (Zeiss, Oberkocken, Germany).

### Immunohistochemical analysis of islets of Langerhans

Pancreatic sections from DR*Lyp*/*Lyp* (*n =* 10, 5M/5F) and control (*n =* 10, 5M/5F) rats were collected on slides and air-dried overnight at 37°C. Slides were deparaffinised [27] and sections incubated with the following primary antibodies overnight at 4°C in moisturising chambers: mouse anti-glucagon (1:9000; G-2654, Sigma Aldrich), guinea pig anti-proinsulin (1:2500; 9003; EuroDiagnostica) and rabbit anti-CD3 (1:200; C7930; Sigma Aldrich). Sections were rinsed in PBS with Triton X-100 for 2 × 10 min. Antibodies for insulin and glucagon was carefully validated as detailed [27, 28]. CD3 specificity was tested using primary antisera pre-absorbed with homologous antigen (100 μg/ml antiserum). Pancreatic sections were incubated with the following secondary antibodies with specificity for mouse, guinea pig, or rabbit IgG: goat anti-mouse Alexa Fluor 568, (1:400; A21124; Invitrogen, Thermo Scientific, Helsingborg, Sweden), goat anti-guinea Pig, Alexa Fluor 594, (1:400; A11076; Thermo Scientific) and goat anti-rabbit, Alexa Fluor 594, (1:400; A11012; Thermo Scientific) [27].

Immunofluorescence was examined in an epi-fluorescence microscope (Olympus, BX60, Tokyo, Japan). By changing filters, double staining was used to determine the location of the different secondary antibodies in one sample. Images were captured with a digital camera (Nikon DS-2Mv, Tokyo, Japan).

### Statistical analysis

Data are expressed as mean ± SEM. IVGTTs, AUC and acute insulin response to glucose (AIR_Glucose_) were calculated as described [6, 29, 30]. Mann–Whitney non-parametrical testing was employed in all experiments, except for analysis of islet size (OPT), blood flow measurements, 1 h batch experiments, insulin content, qPCR and ATP/ADP measurements, which were analysed with Student’s *t* tests, and plasma insulin levels, which were assessed using a two-way ANOVA. Statistical analyses were performed using GraphPad Prism 6 software (GraphPad Software, La Jolla, CA, USA). *p* < 0.05 was considered to be statistically significant. All experiments were performed and analysed in a randomised and blinded fashion when possible. Outliers were identified using Grubbs test for outliers.

## Results

### Diagnosis of diabetes

**Fig. 1:**
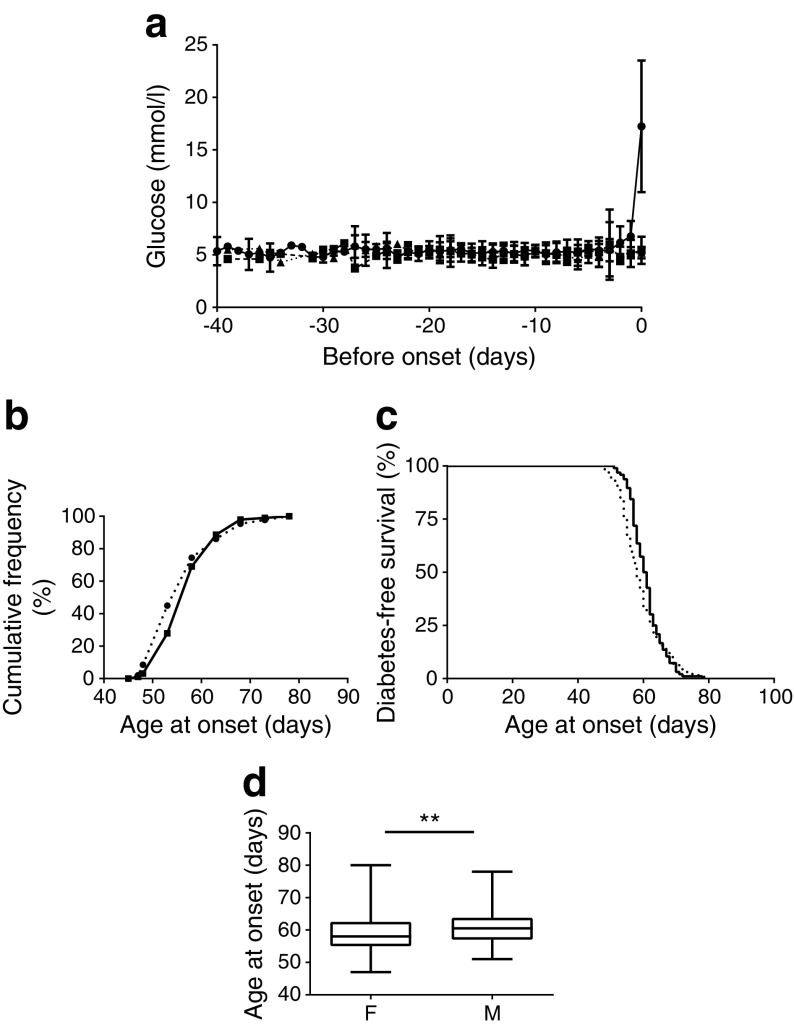
(**a**) Daily glucose levels in 40-day-old female and male DR*Lyp/Lyp* (circles), control (DR*Lyp/+*, triangles and DR*+/+*, squares) rats presented as days before onset of type 1 diabetes. (**b**) Cumulative increase in diabetes incidence in male (solid line, squares) and female (dotted line, circles) DR*Lyp/Lyp* rats. (**c**) Diabetes-free survival in male (solid line) and female (dotted line) DR*Lyp/Lyp* rats. (**d**) Age at onset in female (F) and male (M) DR*Lyp/Lyp* rats. Data shown as means ± SEM. **p < 0.01. DR*Lyp/Lyp*: *n =* 225, 129M/96F; DR*Lyp/+* and DR*+/+*: *n =* 100, 50M/50F

DR*Lyp/Lyp* and control (DR*Lyp/+* and DR*+/+*) rats were followed by daily blood glucose measurements until diagnosis of type 1 diabetes (Fig. 1a). Cumulative incidence revealed that all DR*Lyp/Lyp* rats had developed diabetes by 80 days of age (Fig. 1b). Mean age at onset of type 1 diabetes was 60 days ranging from 47 to 80 days (Fig. 1d). Female rats developed diabetes earlier than males (Fig. 1c; *p =* 0.004).

### Serum insulin prior to type 1 diabetes onset

**Fig. 2:**
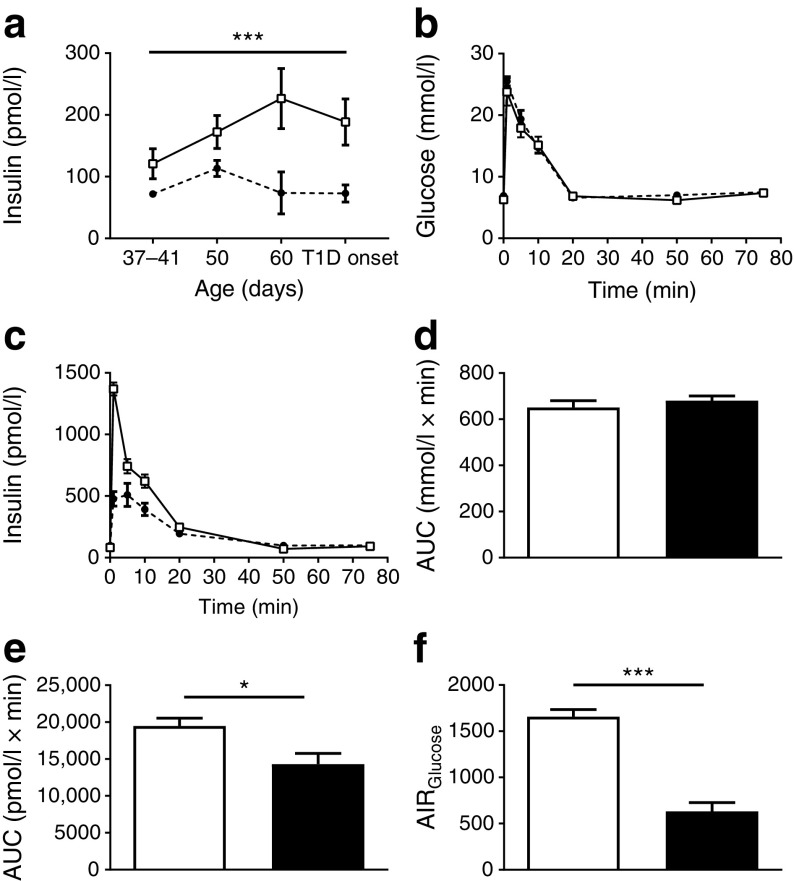
(**a**) Serum insulin over time in DR*Lyp/Lyp* (black circles) and control rats (white squares). At 37–41 days of age: DR*Lyp/Lyp*, *n =* 7 (4M/3F), control, *n =* 10 (5M/5F); at 50 days of age: DR*Lyp/Lyp*, *n =* 6 (3M/3F), control, *n =* 10 (5M/5F); at 60 days of age: DR*Lyp/Lyp*, *n =* 6 (3M/3F), control, *n =* 11 (6M/5F; at type 1 diabetes onset: DR*Lyp/Lyp*, *n =* 7 (4M/3F), control *n =* 9 (5M/4F). (**b**–**f**) IVGTTs in 40-day-old DR*Lyp/Lyp* (black circles/bars; *n =* 10, 6M/4F) and control rats (white squares/bars; *n =* 10, 6M/4F). (**b**) Plasma glucose, (**c**) plasma insulin, (**d**) AUC for glucose and (**e**) AUC for insulin. (**f**) AIR_Glucose_. Data shown as means ± SEM. *p < 0.05, ***p < 0.001. T1D, type 1 diabetes

Basal insulin levels were evaluated in DR*Lyp/Lyp* and control rats over time. Despite normoglycaemia prior to onset of type 1 diabetes, insulin levels were lower at all time points in DR*Lyp/Lyp* rats and failed to increase with age compared with control rats (Fig. 2a; *p =* 0.0004).

### In vivo insulin release is perturbed in DR*Lyp*/*Lyp* rats

In vivo glucose homeostasis and beta cell function were assessed with an IVGTT in DR*Lyp/Lyp* rats. DR*Lyp/Lyp* rats remained glucose tolerant (Fig. 2b). No difference in glucose clearance between groups was observed, also shown as AUC for glucose (Fig. 2d). However, DR*Lyp/Lyp* rats secreted less insulin during the initial time points of the IVGTT vs controls (Fig. 2c) which was further highlighted by a reduction in AUC for insulin in DR*Lyp/Lyp* rats (Fig. 2e; 19466.9 ± 1060.2 vs 14310.8 ± 1454.2 pmol/l × min; *p =* 0.04) and a decrease in the AIR_Glucose_ (Fig. 2f; 1685.3 ± 121.3 vs 633.3 ± 148.7; *p* < 0.0001).

### Insulin secretion is decreased in islets from DR*Lyp*/*Lyp* rats

**Fig. 3:**
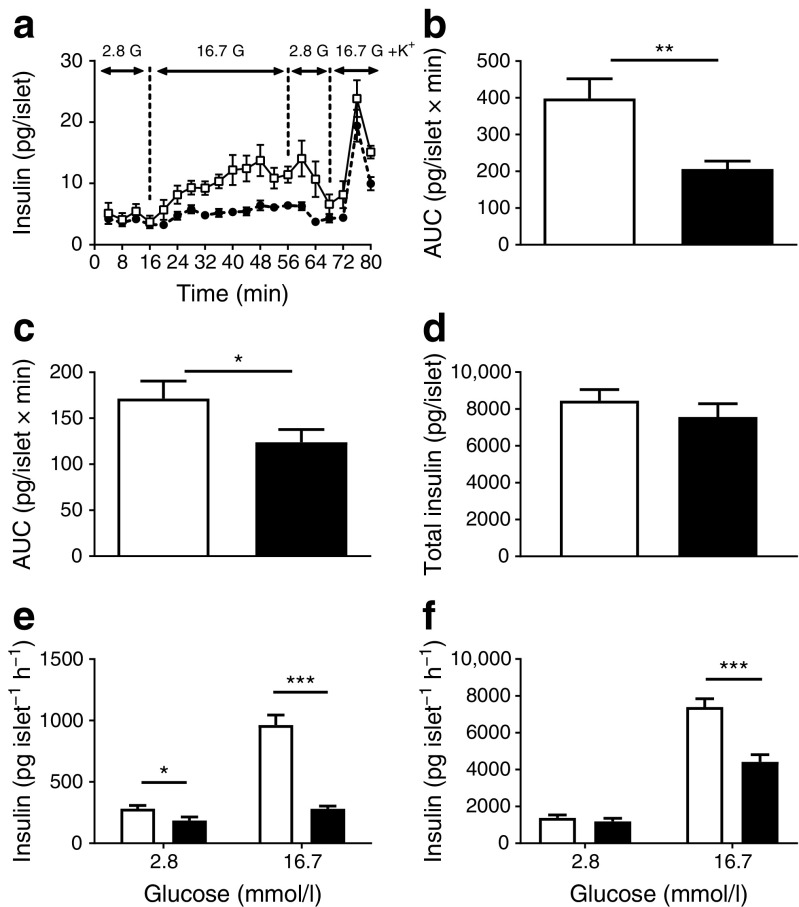
(**a**) Isolated islets from 40-day-old DR*Lyp/Lyp* (black circles; *n =* 14, 9M/5F) and control rats (white squares; *n =* 8, 4M/4Fe) were perifused with 2.8 mmol/l and 16.7 mmol/l glucose (G) with and without 35 mmol/l KCl (K^+^). (**b**–**c**) AUC for secreted insulin (**b**) 16–56 min at 16.7 mmol/l glucose and (**c**) 70–80 min at 16.7 mmol/l glucose + KCl. (**d**) Total insulin content in islets from DR*Lyp/Lyp* (*n =* 6, 3M/3F) and control rats (*n =* 6, 3M/3F). (**e**–**f**) One-hour batch incubation of isolated islets cultured (**e**) over night or (**f**) for 5–7 days. Islets were stimulated with either 2.8 or 16.7 mmol/l glucose. Overnight incubation: DR*Lyp/Lyp*, *n =* 6, (3M/3F), control, *n =* 6 (3M/3F); 5–7 day incubation: DR*Lyp/Lyp*, n *=* 6 (3M/3F), control, *n =* 7 (3M/4F). White bars, control rats; black bars, DR*Lyp/Lyp* rats. Data shown as means ± SEM. *p < 0.05, **p < 0.01, ***p < 0.001

To assess differences in insulin release (as evident by the IVGTT) between DR*Lyp/Lyp* and control rats*,* we characterised the dynamics of insulin secretion in vitro using a perifusion setup. Islets from DR*Lyp/Lyp* and control rats were first subjected to a low concentration of glucose (2.8 mmol/l) (Fig. 3a). Basal insulin secretion was similar between the groups. When challenging islets with a stimulatory concentration of glucose (16.7 mmol/l) during a 40 min period, the amount of insulin secreted by islets from DR*Lyp/Lyp* rats was reduced. Control rats responded robustly to elevated glucose concentrations (Fig. 3b; control vs DR *Lyp/Lyp* AUC: 398.2 ± 53.8 vs 206.1 ± 21.6 pmol/l × min; *p =* 0.002). When islets were further challenged with 35 mmol/l KCl and 16.7 mmol/l glucose for 12 min, islets from DR*Lyp/Lyp* rats continued to secrete less insulin than those from control rats (Fig. 3c; control vs DR *Lyp/Lyp* AUC: 171.5 ± 18.8 vs 123.9 ± 14.9 pmol/l × min; *p =* 0.02). Insulin content, however, was similar in islets from DR*Lyp/Lyp* and control rats (Fig. 3d).

Comparable results to those obtained in perifused islets were observed when islets were exposed to low (2.8 mmol/l) and high (16.7 mmol/l) glucose concentrations during a 1 h static incubation. A reduction both in basal insulin secretion (282.5 ± 59.4 vs 186.0 ± 62.3 pg islet^−1^ h^−1^; *p =* 0.003) and in glucose-stimulated insulin secretion (GSIS; 963.1 ± 162.1 vs 280.3 ± 64.4 pg islet^−1^ h^−1^; *p* < 0.0001) from islets from DR*Lyp/Lyp* rats vs control rats was evident (Fig. 3e). Glucagon secretion was similar in islets from both groups when exposed to low and high glucose concentrations (ESM Fig. 1a).

Previous work suggests that removing islets from an inflammatory milieu can restore GSIS [31]. Therefore, we cultured islets from DR*Lyp/Lyp* and control rats for 5–7 days. Insulin secretion was measured after exposure to low (2.8 mmol/l) and high (16.7 mmol/l) glucose concentrations in a 1 h static incubation. Overall insulin secretion was improved, both in DR*Lyp/Lyp* and control rat islets, but a significant decrease in GSIS was still evident in islets from DR*Lyp/Lyp* rats vs controls (Fig. 3f; 4416.8 ± 1230.5 vs 7393.9 ± 1593.7 pg islet^−1^ h^−1^; *p* < 0.0001).

### *Il1b*, *Ifng* and *Tnf-α* expression in islets isolated from DR*Lyp*/*Lyp* rats

Next we determined expression of cytokines in islets isolated from DR*Lyp/Lyp* and control rats. RNA was extracted either immediately after isolation or after culturing islets for 5–7 days. *Il1b* was present at similar levels in islets just after isolation (ESM Fig. 1b). However, *Tnf*-α and *Ifng* were undetectable. When islets where cultured over a 5–7 day period, detectable levels of all cytokines were present (ESM Fig. 1c) but did not differ between groups.

### Islet blood perfusion

To determine if reduced insulin secretion in vivo was associated with microcirculatory changes [17, 32], we measured islet blood perfusion. Mean arterial blood pressure was recorded in animals prior to blood flow measurements with no significant difference between the two groups (data not shown).

**Fig. 4:**
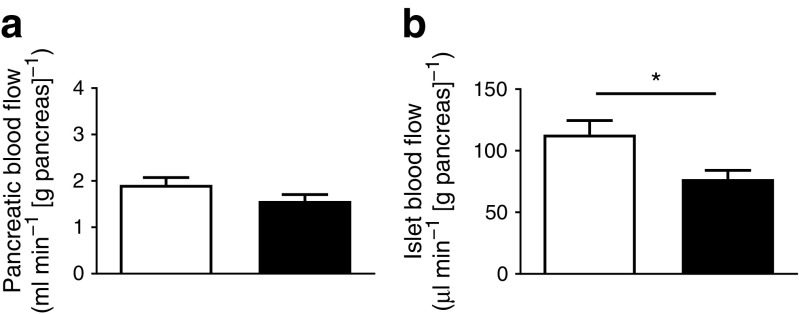
(**a**) Whole pancreatic blood flow and (**b**) islet blood flow in 40-day-old DR*Lyp/Lyp* (*n =* 11, 4M/7F) and control rats (*n =* 15, 6M/9F). White bars, control rats; black bars, DR*Lyp/Lyp* rats. Data shown as means ± SEM. *p < 0.05

Whole pancreatic blood flow did not differ between DR*Lyp/Lyp* and control rats (Fig. 4a). Interestingly, islet blood flow was significantly reduced by 25% in the DR*Lyp*/*Lyp* animals vs controls (Fig. 4b; 76.9 ± 11.8 vs 113.1 ± 16.8 μl min^−1^ [g pancreas]^−1^; *p =* 0.023).

### Small and medium sized islets are less common in the pancreas of DR*Lyp*/*Lyp* rats

**Fig. 5:**
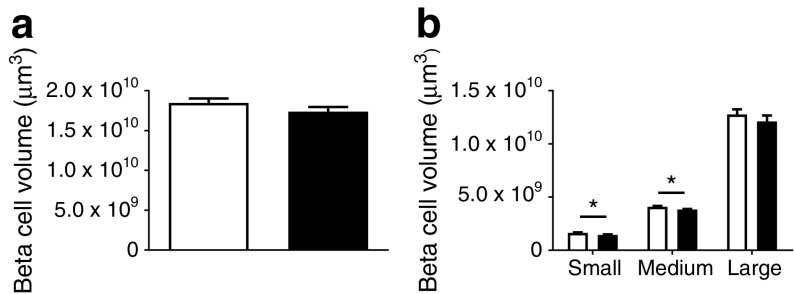
(**a**) Overall beta cell volume in 40-day-old DR*Lyp/Lyp* (*n =* 6, 4M/2F) and control rats (*n =* 4, 2M/2F). (**b**) Islet volumes of arbitrarily chosen islet size categories in DR*Lyp/Lyp* and control rats. White bars, control rats; black bars, DR*Lyp/Lyp* rats. Data shown as means ± SEM. *p < 0.05

**Fig. 6:**
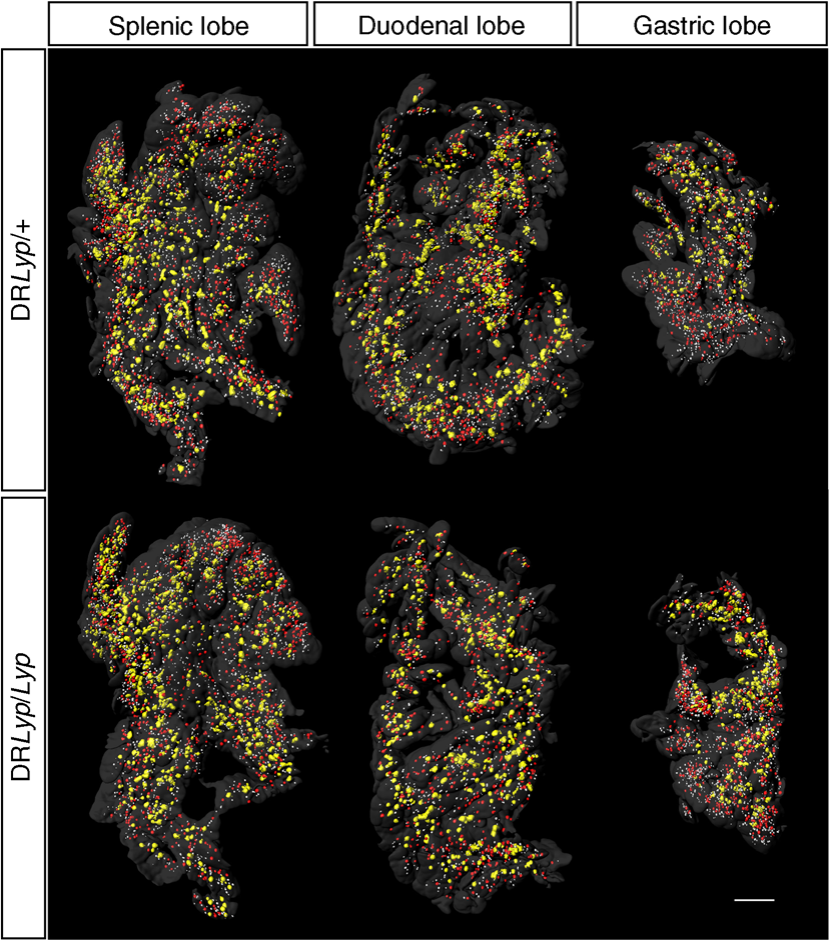
Representative OPT images from a splenic, duodenal and gastric pancreatic lobe from a 40-day-old heterozygote DR*Lyp/+* rat (control) and a DR*Lyp/Lyp* rat. Scale bar, 2 mm

To understand whether the observed perturbation in insulin secretion in vivo was accompanied by differences in beta cell mass, we performed OPT on the whole pancreas from DR*Lyp/Lyp* and control rats. Overall, beta cell mass did not differ between groups (Fig. 5a). However, there was a reduction in small (1.4 × 10^9^ ± 4.5 × 10^7^ vs 1.6 × 10^9^ ± 5.1 × 10^7^ μm^3^; *p =* 0.035) and medium sized islets (3.8 × 10^9^ ± 5.8 × 10^7^ vs 4.1 × 10^9^ ± 9.5 × 10^7^ μm^3^; *p =* 0.044) in the DR*Lyp/Lyp* rats vs control rats (Fig. 5b). Representative images from the OPT of splenic, duodenal and gastric pancreatic lobes from a heterozygote DR*Lyp/+* rat and a DR*Lyp/Lyp* rat (Fig. 6) present size determination by colour coding. Islets were stained with insulin: red depicts large islets, yellow depicts medium sized islets and white depicts small islets. Additionally, we employed a morphometrical method to assess islet mass in our model [22]. We found no decrease in overall islet mass in the DR*Lyp/Lyp* rats compared with controls (ESM Fig. 1d).

### ATP/ADP ratio is increased in islets from DR*Lyp*/*Lyp* rats

**Fig. 7:**
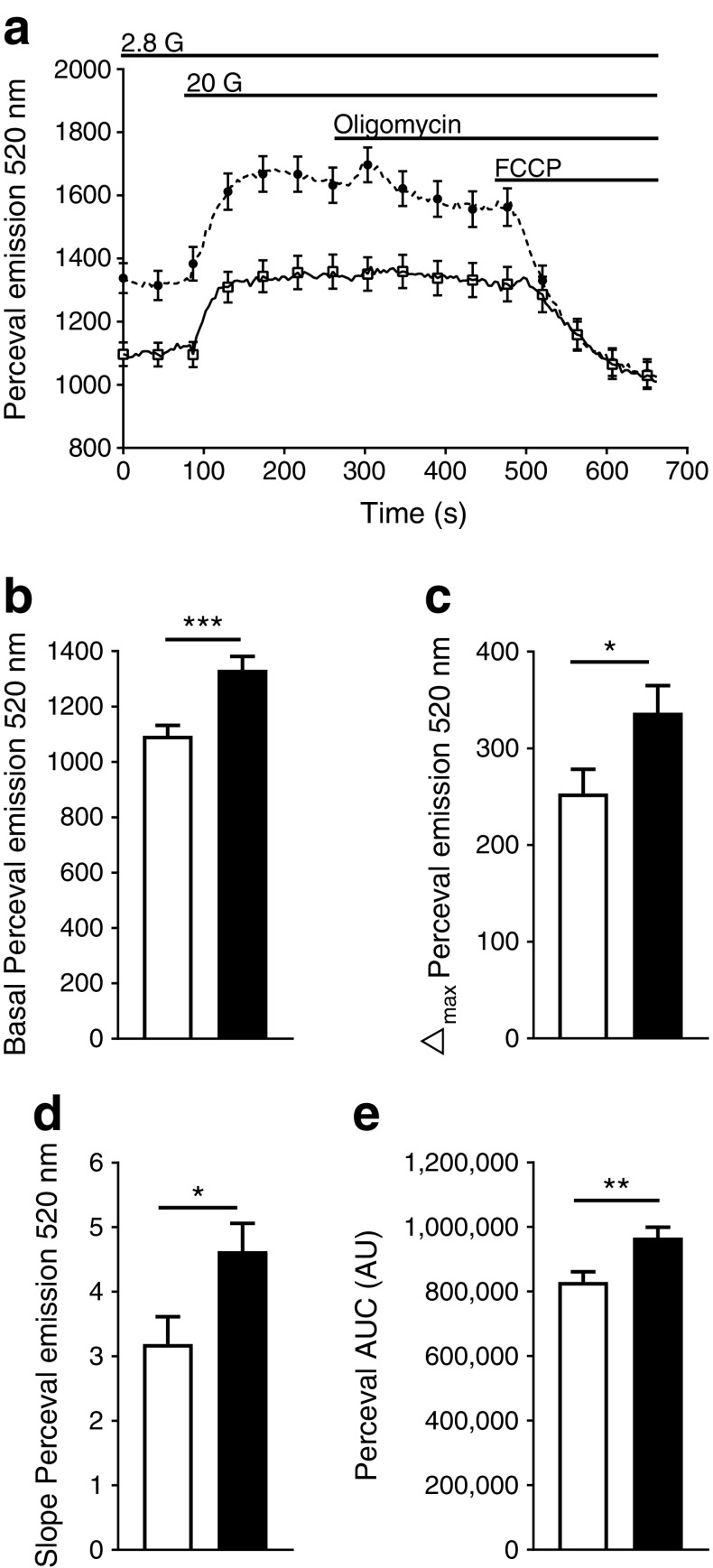
(**a**) ATP/ADP ratio in beta cells from DR*Lyp/Lyp* (black circles; *n =* 91 islets) and control rats (white squares; *n =* 70 islets). (**b**) Basal ATP/ADP ratio, (**c**) Δ_max_ ATP/ADP ratio, (**d**) slope increase of ATP/ADP ratio and (**e**) AUC for ATP/ADP measurements in beta cells from DR*Lyp/Lyp* (black) and control rats (white). Data shown as means ± SEM. *p < 0.05, **p < 0.01, ***p < 0.001. AU, arbitrary units; G, glucose (mmol/l); FCCP, carbonyl cyanide-4-(trifluoromethoxy)phenylhydrazone; G, glucose (mmol/l)

GSIS is dependent on mitochondrial metabolism and the resulting increase in intracellular ratio of ATP/ADP [33]. Therefore, we assessed ATP/ADP ratio in beta cells from DR*Lyp/Lyp* and control rats (Fig. 7a). Interestingly, we observed elevated basal ATP/ADP levels in beta cells from DR*Lyp/Lyp* vs control rats (Fig. 7b; basal Perceval emission at 520 nm: 1333.1 ± 47.3 vs 1094.7 ± 36.4; *p =* 0.0003). Addition of 20 mmol/l glucose raised the ATP/ADP ratio even further in DR*Lyp/Lyp* vs control rats (visualised as Δ_max_ in Fig. 7c; 336.4 ± 31.3 vs 252.2 ± 24.8; *p =* 0.03; and slope-increase in Fig. 7d: 4.6 ± 0.5 vs 3.2 ± 0.4; *p =* 0.02). Moreover, AUC for the whole trace was higher in beta cells from DR*Lyp/Lyp* rats (Fig. 7e; *p =* 0.003). Since mice lacking *Glut2* lose the first phase of insulin secretion [34] and display a similar secretory pattern as our model, we investigated *Glut2* expression in islets from DR*Lyp/Lyp* and control rats. However, expression of *Glut2* was similar in islets from both groups (ESM Fig. 1e).

### Islet morphology and CD3^+^ cells are similar in DR*Lyp*/*Lyp* and control rats

**Fig. 8:**
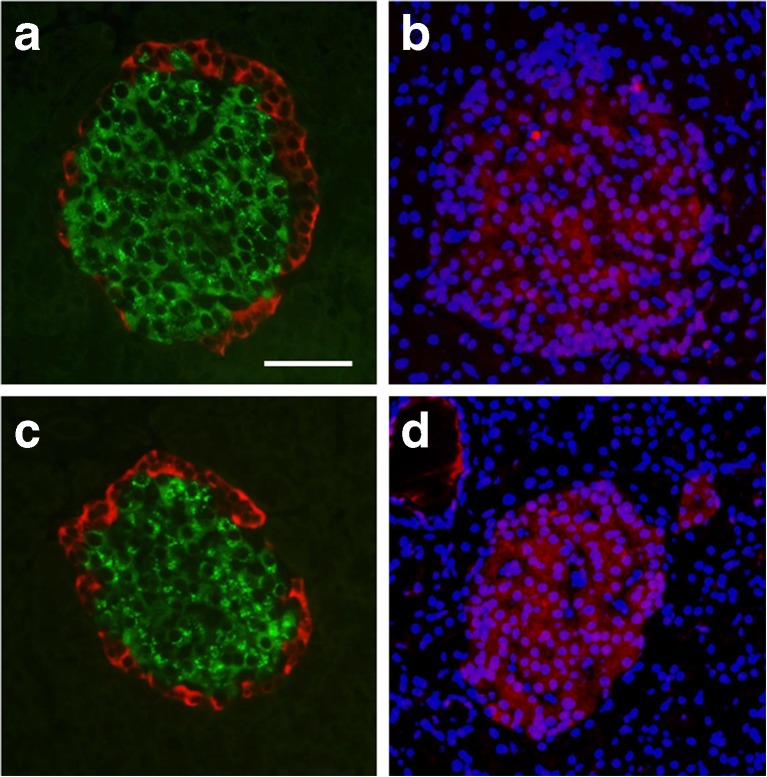
Pancreatic sections from (**a**,**b**) 40-day-old DR*Lyp/Lyp* and (**c**,**d**) control rats (DR*Lyp/+* and DR*+/+*). Sections were stained for (**a**,**c**) insulin (green) and glucagon (red), and (**b**,**d**) CD3^+^ (red) with nuclear DAPI (blue). Scale bar, 50 μm

To determine changes in islet morphology in DR*Lyp/Lyp* rats, we performed insulin and glucagon staining. Islets in pancreatic sections from both DR*Lyp/Lyp* and control rats displayed normal islet architecture (core of beta cells surrounded by alpha cells; Fig. 8a,c). To confirm previous findings that 40-day-old DR*Lyp/Lyp* rats do not present immune cell infiltration, we performed staining using a CD3^+^ specific antibody combined with a nuclear DAPI. As expected, staining was sparse, but similar in DR*Lyp/Lyp* and control animals (Fig. 8b,d).

## Discussion

The present study demonstrates that GSIS is perturbed in the DR*Lyp/Lyp* rat as compared with diabetes-resistant littermates. The secretory defect was accompanied by significant reductions in the number of medium and small sized islets, and reduced intra-islet blood flow. Notably, these islet-specific derangements were observed at 40 days of age before hyperglycaemia, insulitis and onset of type 1 diabetes.

Type 1 diabetes is associated with the immune-mediated destruction of beta cells, resulting in insulin deficiency. Recent advances have highlighted genetic and functional changes within the beta cell as part of type 1 diabetes pathology [4, 29], suggesting that beta cells may have an inherent sensitivity that possibly makes them susceptible to autoimmune attack. We observed a significant reduction in insulin secretion both in vivo and in vitro in isolated islets from DR*Lyp/Lyp* rats. Indeed, a previous study showed that non-inbred BB rats (BB/Hagedorn; a model where lymphopenia is not present) displayed diminished release of insulin during stimulation with 20 mmol/l glucose in perfused whole pancreas at 50 days of age (before onset of type 1 diabetes) [35]. Similar observations have been made in islets from NOD mice, where insulin secretion immediately after isolation was perturbed (due to insulitis). However, culture of islets from NOD mice over a 5–7 day period improved insulin secretion significantly [31]. Indeed, islets from DR*Lyp/Lyp* rats displayed an improved response to glucose after a culturing period; however, a secretory defect was still evident. Similarly, islets removed from people with new-onset type 1 diabetes show improved GSIS after culture [36]. It is noteworthy, however, that GSIS could not be fully restored in all individuals. A major difference between those studies and ours is that insulitis is not present in 40-day-old DR*Lyp/Lyp* rats. Islets from 40-day-old DR*Lyp/Lyp* rats show reduced expression of the complement inhibitor protein CD59. CD59 is pivotal for normal beta cell exocytosis [37], suggesting that beta cell exocytosis is compromised in DR*Lyp/Lyp* rats. This corresponds to our perifusion data, where islets from DR*Lyp/Lyp* rats display an improved response to 35 mmol/l KCl, suggesting that insulin is not lost, rather that exocytosis is compromised. A previous study highlighted similar findings where non-metabolic secretagogues elicit insulin release in prediabetic conditions and in type 1 diabetes [38]. Additionally, insulin content is not altered in isolated islets from 40-day-old DR*Lyp/Lyp* rats, which further supports this notion.

In prediabetic NOD mice, beta cell dysfunction is suggested to occur as a consequence of early immune cell infiltration and activation of inflammatory cascades [39]. However, the DR*Lyp/Lyp* rats do not display any major infiltration by mononuclear cells until a few days prior to clinical onset of type 1 diabetes [13]. We confirmed this, and islets from DR*Lyp/Lyp* rats did not show increased infiltration of CD3^+^ cells in pancreatic sections. Moreover, we were unable to detect elevated expression of *Il1b*, *Ifng* and *Tnf-α* in islets from DR*Lyp/Lyp* rats; cytokines that could be indicative of early immune processes within the islets [40, 41].

Beta cell mass is tightly regulated during fetal life, a time point representing a critical window when the appropriate number of beta cells are set in place [42]. A potential weakness in the present study is that we have not investigated neonatal beta cell growth and postnatal expansion of beta cells in our model. It may very well be that DR*Lyp/Lyp* rats are born with a reduced number of beta cells, or fail to expand their beta cell mass during postnatal stages. We observe significant reductions in small and medium sized islets in DR*Lyp/Lyp* compared with control rats, albeit overall islet mass was not changed. A previous study shows that smaller islets contain more insulin per islet volume in situ and secrete insulin more efficiently in vitro [43]. In addition, large islets may be subjected to both hyperplasia and hypoxia [44], resulting in impaired beta cell function. Thus, loss of small and medium sized islets may very well impact insulin secretion. Additionally, OPT has an advantage over more conventional methods, since it can give information on spatial position and volume of individual insulin-expressing islets throughout the pancreas, with high resolution and the opportunity to categorise islets by size [23].

Another important factor influencing beta cell function is nutritional blood status and islet blood flow. This could be considered as the main avenue by which beta cells are kept informed of the body’s nutritional state [45]. We observed reduced intra-islet blood flow in DR*Lyp/Lyp* rats. The importance of this finding for development of type 1 diabetes remains to be determined, but in general lower blood perfusion in islets could compromise beta cell function through hypoxia or limited dispersal of insulin into the systemic circulation [17, 32]. Moreover, decreased blood flow decreases shear stress, which increases the tendency for leucocyte adhesion in venules even in the absence of additional activators [46]. This could promote islet immune cell infiltration. Indeed, a previous study showed a venular defect in a related rat strain (BB/Wor rat), which supports our findings [18]. Currently, any relationship between blood flow changes and lymphopenia in DR*Lyp/Lyp* rats remains unknown. High basal islet blood flow in diabetes-resistant (and/or wild-type) animals is to a large extent mediated by locally generated nitric oxide from endothelial cells and inhibiting this system decreases blood perfusion [47]. It is noteworthy that studies on islet endothelial cells from young normoglycaemic diabetes-prone and diabetes-resistant BB rats have shown that diabetes-prone rats exhibit considerably lower endothelial cell nitric oxide synthase activity than diabetes-resistant rats [48].

Insulin release is to a large extent dependent on mitochondrial metabolism of glucose and the resulting increase in intracellular ratio of ATP/ADP [33]. Glucose uptake into beta cells is the initial step in GSIS. In rodents this is mediated by GLUT2 [49]. Mice lacking *Glut2* lose the first phase of insulin secretion [34]. Thus, both the ATP/ADP ratio and *Glut2* expression could influence GSIS in DR*Lyp/Lyp* rats. We observed no changes in *Glut2* expression. Intriguingly, however, ATP/ADP levels were elevated in islets from DR*Lyp/Lyp* rats, which could signify a compensatory mechanism as mitochondria are striving to maintain a sufficient ATP/ADP ratio and coupling factors to ensure sufficient insulin release. It may also suggest that the secretory deficiency lies distal of ATP generation (i.e. depolarisation of the plasma membrane/Ca^2+^ influx or exocytosis). Clearly, more intense research efforts are required in this area.

In summary, our results show that DR*Lyp/Lyp* rats display a secretory defect prior to autoimmune onset of type 1 diabetes. This is manifested by perturbations in insulin secretion in vivo and in vitro, partial loss of beta cell mass and reduced intra-islet blood flow; all of which are factors that influence beta cell function. These changes may be of importance for the development of type 1 diabetes.

## Electronic supplementary material

ESM (PDF 90 kb)

## Abbreviations

AIR_Glucose_: Acute insulin response to glucose
BB: BioBreeding
GSIS: Glucose-stimulated insulin secretion
OPT: Optical projection tomography
ROS: Reactive oxygen species
SAB: Secretion assay buffer
