# Supplementary material for: Early deficits in insulin secretion, beta cell mass and islet blood perfusion precede onset of autoimmune type 1 diabetes in BioBreeding rats
Source: Diabetologia. 2017 Dec 6;61(4):896–905. doi: 10.1007/s00125-017-4512-z (PMC6448977; doi:10.1007/s00125-017-4512-z)
Supplement: Supplementary file 1 — (PDF 90 kb) [file 125_2017_4512_MOESM1_ESM.pdf]

ESM Figure 1

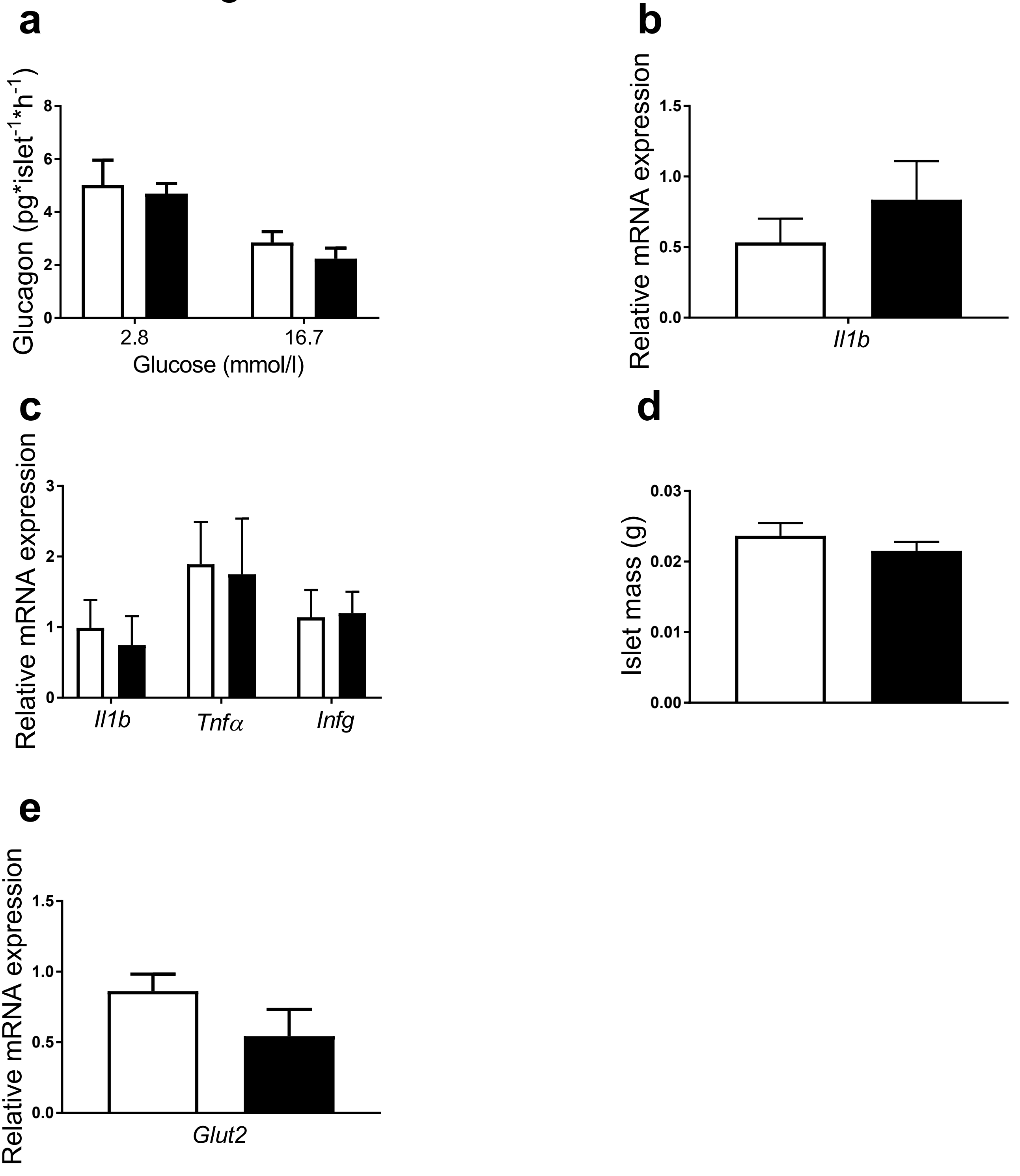

ESM Figure 1. Glucagon secretion from one-hour batch incubations of isolated islets from DRLyp/Lyp (n=6, 3 male and 3 female) rats and control (n=6, 3 male and 3 female) rats stimulated with either 2.8 or 16.7 mmol/l Glucose **(a)**. Expression of *Il1b* in freshly isolated islets from 40-day-old DRLyp/Lyp rats (n=6, 3 male and 3 female) and controls (n=7, 3 male and 4 female), expression of *Tnf-α* and Infg was undetectable **(b)**. Expression of *Il1b*, *Tnf-α* and *Infg* in isolated islets from DRLyp/Lyp rats (n=6, 3 male and 3 female) and controls (n=7, 3 male and 4 female) after culturing islets over a 5-7 day period **(c)**. Islet mass in 40-day-old DRLyp/Lyp rats (n=6, 3 male and 3 female) and controls (n=7, 3 male and 4 female) **(d)**. *Glut2* expression in isolated islets from 40-day-old DRLyp/Lyp rats (n=5, 3 male and 2 female) and controls (n=7, 3 male and 4 female) rats **(e)**. Bar graphs; white bars depict control rats and black bars DRLyp/Lyp rats. Data shown as means ± SEM.
